# In Vitro Characterization of Doxorubicin-Mediated Stress-Induced Premature Senescence in Human Chondrocytes

**DOI:** 10.3390/cells11071106

**Published:** 2022-03-25

**Authors:** Valeria Kirsch, Jan-Moritz Ramge, Astrid Schoppa, Anita Ignatius, Jana Riegger

**Affiliations:** 1 Division for Biochemistry of Joint and Connective Tissue Diseases, Department of Orthopedics, University of Ulm, 89081 Ulm, Germany; valeria.kirsch@uni-ulm.de; 2 Institute for Orthopedic Research and Biomechanics, University of Ulm, 89081 Ulm, Germany; jan-moritz.ramge@uni-ulm.de (J.-M.R.); astrid.schoppa@uni-ulm.de (A.S.); anita.ignatius@uni-ulm.de (A.I.)

**Keywords:** chondrocytes, senescence, SIPS, SASP, oxidative stress, doxorubicin, osteoarthritis, uPAR, ROS, aging

## Abstract

Accumulation of senescent chondrocytes is thought to drive inflammatory processes and subsequent cartilage degeneration in age-related as well as posttraumatic osteoarthritis (OA). However, the underlying mechanisms of senescence and consequences on cartilage homeostasis are not completely understood so far. Therefore, suitable in vitro models are needed to study chondrocyte senescence. In this study, we established and evaluated a doxorubicin (Doxo)-based model of stress-induced premature senescence (SIPS) in human articular chondrocytes (hAC). Cellular senescence was determined by the investigation of various senescence associated (SA) hallmarks including β-galactosidase activity, expression of p16, p21, and SA secretory phenotype (SASP) markers (IL-6, IL-8, MMP-13), the presence of urokinase-type plasminogen activator receptor (uPAR), and cell cycle arrest. After seven days, Doxo-treated hAC displayed a SIPS-like phenotype, characterized by excessive secretion of SASP factors, enhanced uPAR-positivity, decreased proliferation rate, and increased β-galactosidase activity. This phenotype was proven to be stable seven days after the removal of Doxo. Moreover, Doxo-treated hAC exhibited increased granularity and flattened or fibroblast-like morphology. Further analysis implies that Doxo-mediated SIPS was driven by oxidative stress as demonstrated by increased ROS levels and NO release. Overall, we provide novel insights into chondrocyte senescence and present a suitable in vitro model for further studies.

## 1. Introduction

Osteoarthritis (OA) is the most common joint disease in the world, primarily affecting the elderly population [1]. In general, OA is associated with detrimental changes in cartilage metabolism, characterized by an imbalance between the breakdown and biosynthesis of extracellular matrix (ECM) components, resulting in a progressive degeneration of the tissue [2]. After several years, this ultimately leads to bone–bone contact, causing symptoms such as pain, swelling, and impaired joint mobility [3]. Although aging is the biggest risk factor for the development of chronic diseases such as OA, further determinants including genetic predisposition and extensive mechanical stress can accelerate the progression of OA [4,5].

Cellular senescence has primarily been described as cell cycle arrest in proliferating cells [6,7]. Despite the post-mitotic nature of chondrocytes, senescence is regarded as a crucial molecular mechanism and potential driver in age- and injury-related OA [3,8]. Interestingly, Xu et al. demonstrated that the transplantation of senescent fibroblasts into the knee joints of healthy mice resulted in an OA-like phenotype [9]. This study, amongst others, suggests that senescent cells (snCs) negatively affect the structure and function of adjacent tissues and cells, implying a spreading of senescence [3]. This phenomenon can be attributed to the secretion of pro-inflammatory factors, a typical hallmark of snCs, which is known as the senescence-associated secretory phenotype (SASP). 

Due to the accumulation of snCs in the cartilage of OA patients, SASP factors including cytokines (IL-6, IL-1ß), chemokines (CXCL1), and matrix-degrading enzymes such as proteases (MMP-13) are increasingly secreted [10,11]. These pro-inflammatory factors are capable of inducing a chronic low-grade inflammation, a process referred to as ‘inflammaging’, leading to an increase in reactive oxygen species (ROS) [12]. The enhanced levels of ROS, in turn, cause oxidative stress, which leads to the activation of redox-sensitive pathways and subsequent expression of catabolic and pro-inflammatory mediators [13]. Moreover, oxidative stress results in the damage of DNA, proteins, and lipids [12]. Consequently, the progression of senescence is driven by the inflammatory microenvironment and cellular damage [14]. Amongst the secretion of SASP, senescent chondrocytes lose their typical phenotype and acquire hypertrophic and fibroblastic features [15].

Besides aging, irradiation, and trauma, certain chemotherapeutic drugs such as doxorubicin (Doxo) are potential inducers of senescence [16]. In the case of cartilage injury, chondrosenescence is primarily triggered by an increased generation of ROS and subsequent DNA damage, causing a so-called stress-induced premature senescence (SIPS) [3,17]. Equivalent to the mechanisms observed in replicative senescence, ROS-associated SIPS has been linked to telomere shortening. However, in contrast to replicative senescence, SIPS has also been found to occur independently of telomere shortening [18,19]. Doxo stimulation is generally an established method for the investigation of senescence in various cell types in vitro [20,21,22]. Nonetheless, there have been no profound studies that have described the phenotypical alterations of primary cells, and in particular, that of chondrocytes, after Doxo stimulation in detail. Therefore, the aim of this study was to gain a better insight into Doxo-induced SIPS in human articular chondrocytes (hACs). Moreover, we intended to clarify the irreversibility of senescence in hACs after the removal of Doxo. This represents a key criterion concerning the suitability of the Doxo in vitro model for studying chondrosenescence, since presence of the drug might affect the outcome.

Overall, we could confirm that low concentrations of Doxo induce a senescence-like phenotype in hAC, exhibiting typical hallmarks of senescence such as a low proliferation rate (cell cycle arrest), enhanced SA-β-galactosidase (SA-β-gal) activity, uPAR positivity, and secretion of SASP factors. Analysis of intracellular ROS levels and NO release indicated that Doxo-induced senescence was linked to oxidative stress. Moreover, the senescent phenotype remained stable for at least seven days after deprivation of Doxo. Taken together, our study provides a detailed characterization of SIPS in hAC and evidence for the suitability of the Doxo-based in vitro model.

## 2. Materials and Methods

### 2.1. Isolation and Cultivation of hAC

Human cartilage was obtained from OA patients undergoing total knee joint replacement. Informed consent was obtained from all patients according to the terms of the Ethics Committee of the University of Ulm (ethical approval number 353/18). hAC were enzymatically isolated from macroscopically intact (OARSI grade ≤1) tissue [23]. In short, full-thickness cartilage was minced and digested for 45 min with 0.2% pronase (Sigma-Aldrich, Taufkirchen, Germany) and overnight with 0.025% collagenase (Sigma-Aldrich). After washing with PBS and filtration through a 40 μm cell strainer, cells were cultured in serum-containing chondrocyte medium (1:1 DMEM 1 g/L glucose and Ham’s F12, 10% fetal bovine serum (FBS), 0.5% penicillin/streptomycin, 2 mM L-glutamine, and 10 μg/mL 2-phospho-L-ascorbic acid trisodium salt). hAC were split at a confluence of 80% and used at passages 0 to 2.

### 2.2. Stimulation of Isolated hAC

SIPS was induced by stimulation with different concentrations of doxorubicin (Doxo; Selleckchem, Houston, TX, USA) for 5 d, 7 d, or 10 d in a serum-reduced chondrocyte medium (5% FBS). Doxo was refreshed concomitantly with medium changed twice a week. Induction of apoptosis was attained by stimulation with cycloheximide (CHX; concentration: 10 µg/mL; Sigma-Aldrich) and tumor necrosis factor alpha (TNFa; concentration: 10 ng/mL; Peprotech, Hamburg, Germany) as previously reported [24]. 

### 2.3. Alamar Blue Assay (Cytotoxicity/Cell Proliferation Assay)

Quantitative measurement of cell proliferation/cytotoxicity was attained by means of an Alamar Blue assay (BioRad, Munich, Germany). The conversion of non-fluorescent resazurin to fluorescent resorufin during cellular respiration can be considered as proportional to the number of living cells. After 48 h, wells were rinsed with PBS and cells were incubated for 3 h in 200 µL of a 5% Alamar Blue solution (in serum-free medium) at 37 °C. After the incubation, the fluorescence intensities were detected at a 550 nm excitation and 590 nm emission by using the multimode microplate reader Infinite M200 Pro (Tecan Deutschland, Crailsheim, Germany). Blank values (5% Alamar Blue solution in empty well) were subtracted from the measured values. Unstimulated cells served as the baseline (100% cell viability).

### 2.4. Caspase-3/7 Activity Assay

Activation of the caspase cascade was analyzed using the Amplite™ fluorimetric caspase-3/7 Assay Kit according to the manufacturer’s protocols (AAT Bioquest, Sunnyvale, CA, USA). In short, cells were seeded on a 96-well plate (20,000 cells per well). The next day, cells were stimulated with different concentrations of Doxo for 6.5 h at 37 °C. Cells stimulated with cycloheximide (10 µg/mL) and TNF (10 ng/mL) served as the positive control. Afterward, the caspase-3/7 working solution was added and incubated for 2 h at room temperature in the dark. After centrifugation of the plate, fluorescence was measured on a black bottom 96-well plate at 350 nm excitation and 450 nm emission using an infinite M200 PRO TECAN reader.

### 2.5. SA-β-Galactosidase (SA-β-gal) Staining 

SA-β-gal staining was performed using a SA-β-gal Staining Kit according to the manufacturer’s protocols (Cell Signaling Technology, Danvers, MA, USA). In short, cells were seeded on chamber slides (5000 cells/cm^2^) and cultivated overnight. The next day, cells were fixed in a 2% formaldehyde and 0.2% glutaraldehyde solution for 15 min. After washing with PBS, cells were stained overnight in an X-gal staining solution at 37 °C (dry incubator; low CO_2_). Blueish stained cells were considered as SA-β-gal positive.

### 2.6. Quantitative Real-Time PCR (qRT-PCR)

mRNA of least 50,000 hAC was isolated using a RNeasy Mini Kit (Qiagen, Hilden, Germany). RNA was reverse transcribed with the Superscript II Kit (ThermoFisher, Schwerte, Germany) and used for quantitative real-time PCR analysis (StepOne-PlusTM Real-Time PCR System; Applied Biosystems, Darmstadt, Germany). Determination of the relative expression levels was performed by means of the ∆∆ Ct method. GAPDH, HPRT1, and TMEM199 served as housekeeping genes [20]. TaqMan Gene Expression Assays (ThermoFisher) used in this study: CAT (Hs00156308); CDKN1A (Hs00355782); CDKN2A (P16INK4/P14ARF; Hs00923894); CXCL1 (Hs00605382); GAPDH (Hs02758991); HPRT1 (Hs02800695); IL6 (Hs00985639); IL8 (Hs00174103); iNOS (Hs01075529); MMP13; NOX2/CYBB (Hs00166163); NOX4 (Hs00418351); SIRT (Hs01009006); SOD1 (Hs00533490); SOD2 (Hs00167309); and TMEM199 (Hs01022209). 

For quantification of the telomere length, DNA of 1 × 10^5^ cells was isolated using a DNeasy Blood and Tissue Kit (Qiagen), followed by a qPCR using the Relative Human Telomere Length Quantification Assay Kit (ScienCell, San Diego, CA, USA) according to the manufacturer’s protocols. 

### 2.7. Analysis of Culture Media

Culture media was harvested after 5 d and 10 d, respectively. In both cases, the last media change was performed 48 h before. Quantification of proteins was performed by means of the following enzyme-linked immunosorbent assay (ELISA) kits according to the manufacturer’s instructions: secreted MMP-13 was determined using the human Quantikine ELISA Kit (Ray-Biotech, Peachtree Corners, GA, USA) and in the case of IL-6 and IL-8, human IL-6 and IL-8 uncoated ELISAs (Invitrogen, Carlsbad, CA, USA) were used. NO levels were determined by quantification of nitrite using a Griess assay (Griess Reagent System; Promega, Waldorf, Germany). The total amounts of the specific proteins (MMP-13, IL-6, or IL-8) and NO were normalized to the relative fluorescence intensities of the Alamar Blue assay, which approximates the number of living cells.

### 2.8. Flow Cytometric Analysis

hAC were detached using PBS-buffered EDTA (5 mM) and either stained with anti-uPAR (Invitrogen; MA5-28588) or the corresponding isotype control (BD Bioscience, Heidelberg, Germany) on ice, for 30 min in the dark. A minimum of 3 × 10^4^ cells were analyzed on a Becton Dickinson FACSCalibur flow cytometer (BD Biosciences) with dual-laser technology and the corresponding software CellQuest (BD Biosciences, Version 5.2.1). The percentage of positively stained cells was calculated as uPAR-positive cells minus the isotype control. Overall, a maximum of 1% isotype control-positive cells was tolerated. Explanation of the corresponding gating strategy is provided as Supplementary Figure S1. 

In case of the cell cycle analysis, 1 × 10^6^ cells were trypsinated, washed, and stained in 1 mL PBS containing 10 µM of the Vybrant™ DyeCycle™ Green Stain dye (Invitrogen) at 37 °C for 30 min. At least 250,000 cells/approach were counted.

### 2.9. Immunofluorescence Staining

hAC were fixed with formalin, permeabilized with 0.1% PBS-Tween 20, and incubated for 1 h at 37 °C with blocking buffer (Agilent Technologies, Waldbronn, Germany). Afterwards, cells were stained with anti-CDKN2A (Abcam, Cambridge, UK; ab108349; 1:250) for 2 h at RT, followed by an incubation with a biotinylated link antibody (Agilent Technologies) and another incubation with iFluor568-conjugated avidin (ATT Bioquest), each for 20 min at RT. Nuclei were counterstained with 0.25 µg/mL Dapi for 15 min.

### 2.10. DCFDA and MitoSOX Assay

Analysis of cytoplasmatic ROS levels was performed by means of the DCFDA/H2DCFDA-Cellular ROS Assay Kit (Abcam). In short, cultured hAC were incubated with a 1 µM DCFDA working solution for 45 min at 37 °C. Afterward, samples were analyzed with a fluorescence microscope. Area, fluorescence, and integrated density of the cells were measured with Fiji (Version 2.1.0/1.53c; open-source software). To discriminate between specific and unspecific signals, the mean fluorescence of the untreated hAC was calculated and then subtracted from the measured fluorescence of each cell. Then, the corrected total cell fluorescence (CTCF) was determined for the DCFA-positive cells.

For the co-staining of mitochondrial superoxide, cells were first stained using the DCFDA assay as described above, followed by a staining with the MitoSOX™ Red Mitochondrial Superoxide Indicator (Invitrogen). Cells were incubated in a 5 µM working solution at 37 °C for 10 min.

### 2.11. Western Blot Analysis

For protein isolation, hAC were washed with ice-cold PBS, centrifuged at 1200 rpm for 5 min, and resuspended in ice-cold cell lysis buffer (PierceTM RIPA Buffer; Thermo Scientific, Waltham, MA, USA). The cell lysates were pipetted several times with a syringe, stored on ice for 10 min, and then centrifuged at 14,000 rpm for 10 min at 4 °C. Afterward, the supernatant was transferred into a new tube and the protein concentration was determined by a BCA assay (Thermo Scientific #23227) according to the manufacturer’s instructions. Three times sample buffer and 10 µL loading dye (sample buffer with 5% β-mercaptoethanol) were added to 20 µg protein/sample, boiled at 95 °C for 15 min, and then loaded on SDS-PAGE for protein separation. Samples were transferred onto a PDVF membrane and detected by using Sirt1 (NBP1-51641SS, 1:1000; Novusbio, Littleton, CO, USA), Sod2 (StressMarq, SPC-118, 1:1000), or α-Tubulin (Cell Signaling, #2125, 1:1000) antibodies. After incubation with HRP-linked secondary antibodies, the proteins were visualized with the BioRad ChemiDoxTM MP Imaging system.

### 2.12. Statistical Analysis

Experiments were analyzed using GraphPad Prism8 (GraphPad Software, Inc., San Diego, CA, USA). Datasets with n ≥ 5 were tested for outliers by means of the Grubbs’ outlier test. Outliers were not included in the statistical analyses. Each data point represents an independent biological replicate (donor). Further information about the applied statistical analysis is provided in the respective figure legend. In each case, significance level was set to α = 0.05.

## 3. Results

### 3.1. Doxo Stimulation Results in Cell Cycle Arrest or Apoptosis of hAC

**Figure 1:**
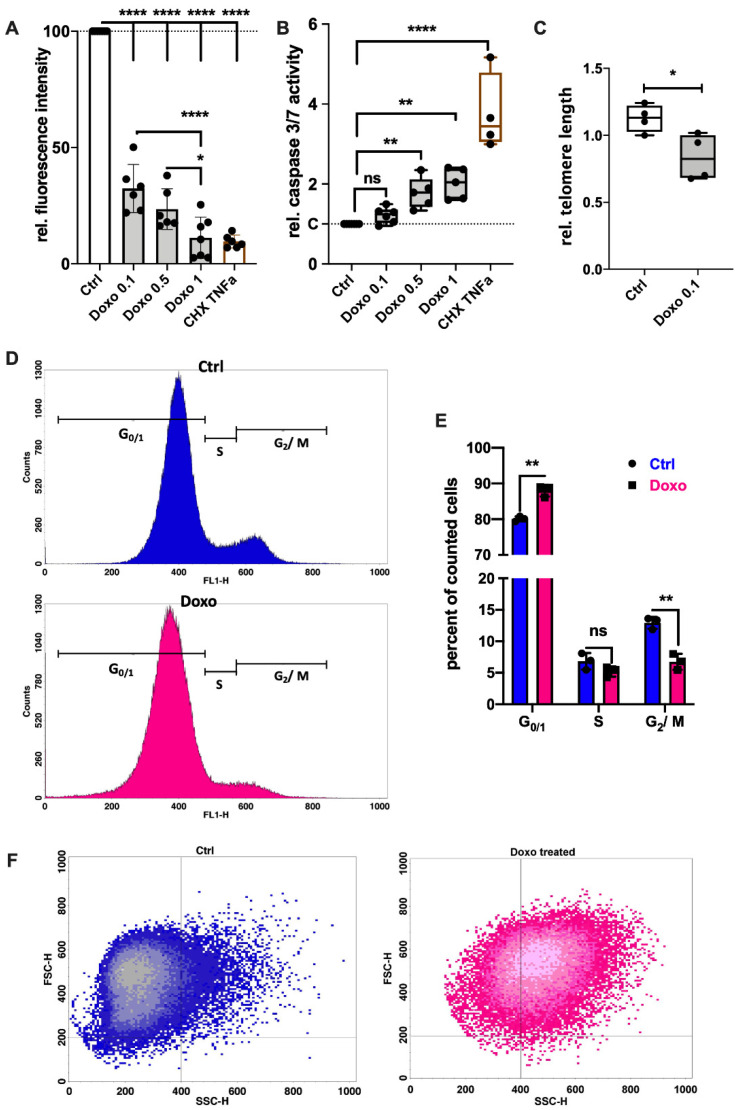
Influence of Doxo stimulation on the cell cycle and apoptosis of hAC. (**A**) Fluorescence intensity of the Alamar Blue assay after 7 d of Doxo-stimulation relative to the unstimulated control; TNFa + CHX served as the apoptosis control; n ≥ 6. (**B**) Relative caspase-3/7 activity after the addition of Doxo at different concentrations or TNF + CHX (apoptosis control); n ≥ 5. (**C**) Relative telomere length in untreated hAC compared to hAC treated with 0.1 µM Doxo for 7 d. (**D**) Exemplary histogram of the flow cytometric cell cycle analysis; blue curve = untreated hAC at passage 2; pink curve = hAC at passage 2, treated with 0.1 µM Doxo for 7 d. (**E**) Corresponding statistics of the cell cycle analysis; n = 3. (**F**) Exemplary dot plots of the cell size (FSC-H) vs. granularity (SSC-H) of unstimulated (blue) or Doxo-stimulated (pink) hAC, assessed by flow cytometry. Data are presented as scatter plot with bars, mean with standard deviation; or box plots with median, whiskers min to max. Significant differences between groups are depicted as: **** *p* ≤ 0.0001; ** *p* ≤ 0.01; * *p* ≤ 0.05. Statistical analysis: (**A**,**B**) one-way ANOVA, Sidak’s multiple comparisons test; (**C**) paired *t*-test; (**D**) multiple *t*-test. Ctrl = control (unstimulated cells), Doxo = doxorubicin, rel. = relative, ns = not significant.

Doxo has been described as an inducer of SIPS in various cell types including chondrocytes [16,25]. Since high doses of Doxo might also lead to apoptotic cell death, both caspase-3/7 activity and metabolic activity were determined at different concentrations ranging from 0.1 µM to 1 µM. Despite a significant decline in the measurable metabolic activity in the Alamar Blue assay after 7 d (Figure 1A), Doxo stimulation did not induce detectable caspase activity at a concentration of 0.1 µM (Figure 1B). In contrast, Doxo concentrations > 0.1 µM significantly enhanced the caspase activity. Measurement of the telomere length revealed that 0.1 µM Doxo treatment for 7 d significantly decreased the relative telomere length in hAC compared to the untreated control (*p* = 0.026; Figure 1C). 

Further analysis of the cell cycle (Figure 1D,E) confirmed a significant reduction in the G_2_/M phase by about 6% (Ctrl: 12.9%; Doxo: 6.7%; *p* = 0.002) in hAC treated with 0.1 µM Doxo for 7 d, while the amount of cells in the G_0/1_ phase was enhanced by about 8% (Ctrl: 80.1%; Doxo: 87.9%; *p* = 0.0015). Moreover, flow cytometric analysis revealed a significant increase in granularity in Doxo-stimulated hAC (Figure 1F). Accordingly, unstimulated cells exhibited an SSC-H < 400, while the majority of Doxo-stimulated cells were found to have an SSC-H > 400.

### 3.2. Doxo Stimulation Increases the Expression of Key Senescence Regulators and SASP

**Figure 2:**
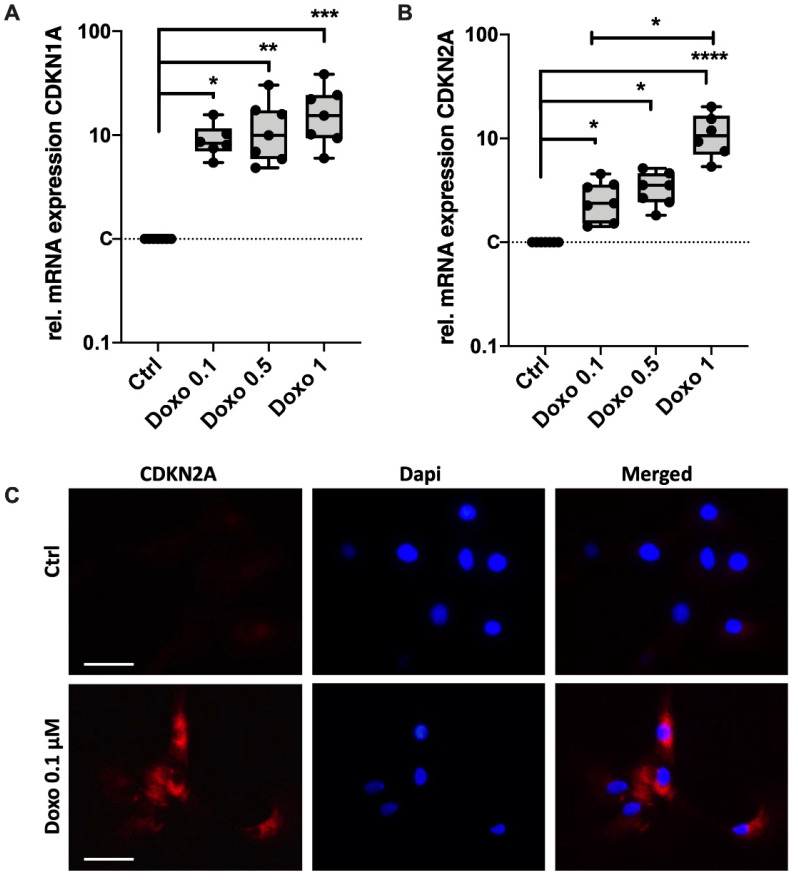
Effect of Doxo stimulation on the expression of key senescence regulators CDKN1A and CDKN2A. Gene expression analysis of (**A**) CDKN1A (p21) and (**B**) CDKN2A (p16) in hAC after 10 d of Doxo stimulation at different concentrations; unstimulated hAC served as controls. (**C**) Exemplary images of a CDKN2A immunostaining in hAC after stimulation with 0.1 µM Doxo for 7 d. Data are presented as box plots with median, whiskers min to max. Significant differences between groups are depicted as: **** *p* ≤ 0.0001; *** *p* ≤ 0.001; ** *p* ≤ 0.01; * *p* ≤ 0.05. Statistical analysis: (**A**,**B**) One-way ANOVA, Sidak’s multiple comparison. Scale bar = 50 μm. Ctrl = control (unstimulated cells), dapi = 4′,6-diamidino-2-phenylindole.

Induction of SIPS in Doxo-stimulated chondrocytes was further confirmed by means of gene expression analysis of the key senescence regulators CDKN1A (p21^Cip1^) and CDKN2A (p16^Ink4a^). mRNA levels of CDKN1A and CDKN2A were significantly enhanced after stimulation with 0.1 µM Doxo by 9.2-fold (*p* = 0.03) and 2.7-fold (*p* = 0.05), respectively (Figure 2A,B). While higher concentration of Doxo had no enhancing effect on the mRNA levels of CDKN1A, significant differences were found between 0.1 µM and 1 µM in the case of CDKN2A (*p* = 0.038). Increased expression of CDKN2A was confirmed by means of exemplary immunostaining in hAC after 10 d of Doxo stimulation (Figure 2C).

**Figure 3:**
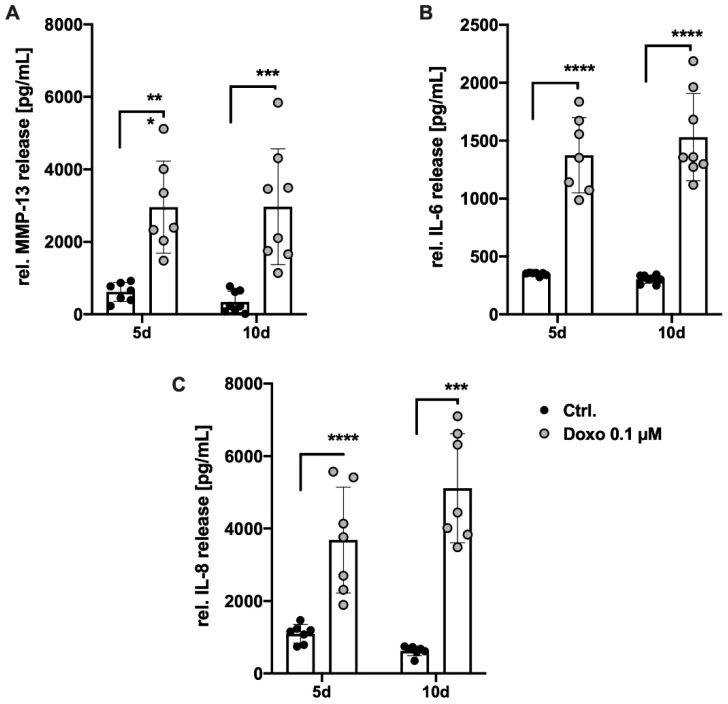
Influence of Doxo stimulation on the release of SASP factors in hAC. Secretion of (**A**) MMP-13, (**B**) IL-6, and (**C**) IL-8 into the culture media after 5 d and 10 d of Doxo stimulation was quantified by means of specific ELISAs. Data are presented as scatter plot with bars, mean with standard deviation. Significant differences between groups are depicted as: **** *p* ≤ 0.0001; *** *p* ≤ 0.001; ** *p* ≤ 0.01; * *p* ≤ 0.05. Statistical analysis: (**A**–**C**) Multiple *t*-test. Ctrl = control (unstimulated cells), rel. = relative.

Aside from the expression of CDKN1A and CDKN2A, cellular senescence is characterized by enhanced secretion levels of catabolic and pro-inflammatory mediators—referred to as the SASP. After 5 d, culture media analysis of Doxo-stimulated hAC revealed a significant increase in the secreted amount of MMP-13 (4.8-fold; *p* = 0.0004), IL-6 (3.9-fold; *p* < 0.0001), and IL-8 (3.4-fold; *p* < 0.0001) compared to the unstimulated control (Figure 3A–C). Prolonged stimulation time with Doxo for 10 d did not result in significant enhancement of the respective protein concentrations. Together, it can be concluded that a dose of 0.1 µM Doxo administered for at least 5 d effectively induced SASP in hACs.

### 3.3. Doxo-Mediated SIPS Results from Intracellular ROS Accumulation 

**Figure 4:**
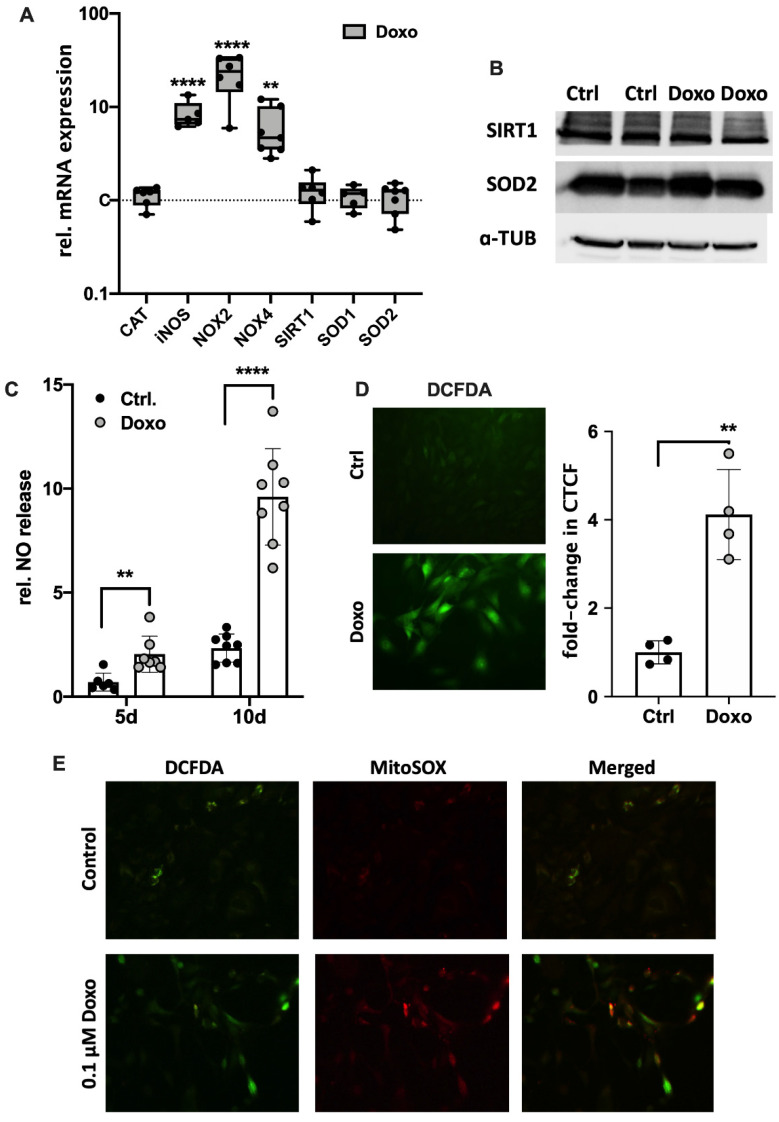
Effects of Doxo stimulation on oxidative stress markers in hAC. (**A**) Gene expression of iNOS, NOX2, and NOX4 as well as CAT, SIRT1, SOD1, and SOD2 after Doxo stimulation for 10 d. (**B**) Exemplary western blot analysis of SIRT1 and SOD2 after Doxo stimulation (n = 2); a-TUB = alpha tubulin. NO release into culture media of Doxo-treated hAC after 5 d and 10 d, respectively. (**D**) Representative images of DCFDA staining of unstimulated and Doxo-stimulated hAC after 7 d and corresponding quantification of the corrected total cell fluorescence (CTFC). (**E**) Exemplary co-staining of mitochondrial superoxide (MitoSOX; red) and cytoplasmic ROS (DCFDA; green) of unstimulated (Ctrlol) and Doxo-stimulated hAC after 7 d. Ctrl = unstimulated hAC. Data are presented as scatter plot with bars, mean with standard deviation; or box plots with median, whiskers min to max. Significant differences between groups are depicted as: **** *p* ≤ 0.0001; ** *p* ≤ 0.01. Statistical analysis: (**A**) One-way ANOVA, Sidak’s multiple comparison; (**C**) multiple *t*-test; (**D**) paired *t*-test. Ctrl = control (unstimulated cells), rel. = relative.

ROS accumulation and subsequent oxidative stress is considered as a driver of cellular senescence. Therefore, the gene expression of ROS-generating NADPH oxidases (NOX2 and NOX4) and inducible nitric oxide synthase (iNOS) as well as intracellular enzymes of the antioxidant system including catalase (CAT), superoxide dismutase 1 (SOD1; cytosolic) and 2 (SOD2; mitochondrial) as well as their regulator sirtuin 1 (SIRT1), was determined by means of qRT-PCR. While mRNA levels of iNOS, NOX2, and NOX4 were significantly increased in Doxo-stimulated hAC (iNOS: 8.5-fold, *p* ≤ 0.0001; NOX2: 23-fold, *p* = 0.0002; NOX4: 6-fold; *p* = 0.003), no alteration was found in the gene expression of CAT, SIRT1, SOD1, or SOD2 (Figure 4A). However, the positive trend in the gene expression of SIRT1 and SOD2 (both 1.3-fold) in the Doxo-stimulated hAC was also observed on the protein level (Figure 4B and Figure S2). In line with the gene expression levels, culture media analysis confirmed enhanced NO release in the presence of Doxo, which was increased with prolonged stimulation time (Figure 4C; 5 d: 3-fold; *p* = 0.006; 10 d: 4-fold; *p* ≤ 0.0001). Determination of cytoplasmic ROS using a DCFDA assay revealed a 4.1-fold increase in the fluorescence intensities of Doxo-stimulated hAC relative to the unstimulated control, confirming enhanced oxidative stress levels (*p* = 0.005; Figure 4D). Moreover, exemplary co-staining of cytoplasmic ROS (DCFDA assay) and mitochondrial superoxide (MitoSox) revealed that Doxo stimulation increased mitochondrial ROS generation (Figure 4E).

### 3.4. Doxo Stimulation Leads to Continuing Expression of SA Markers in hAC Even after Deprivation of the Drug

**Figure 5:**
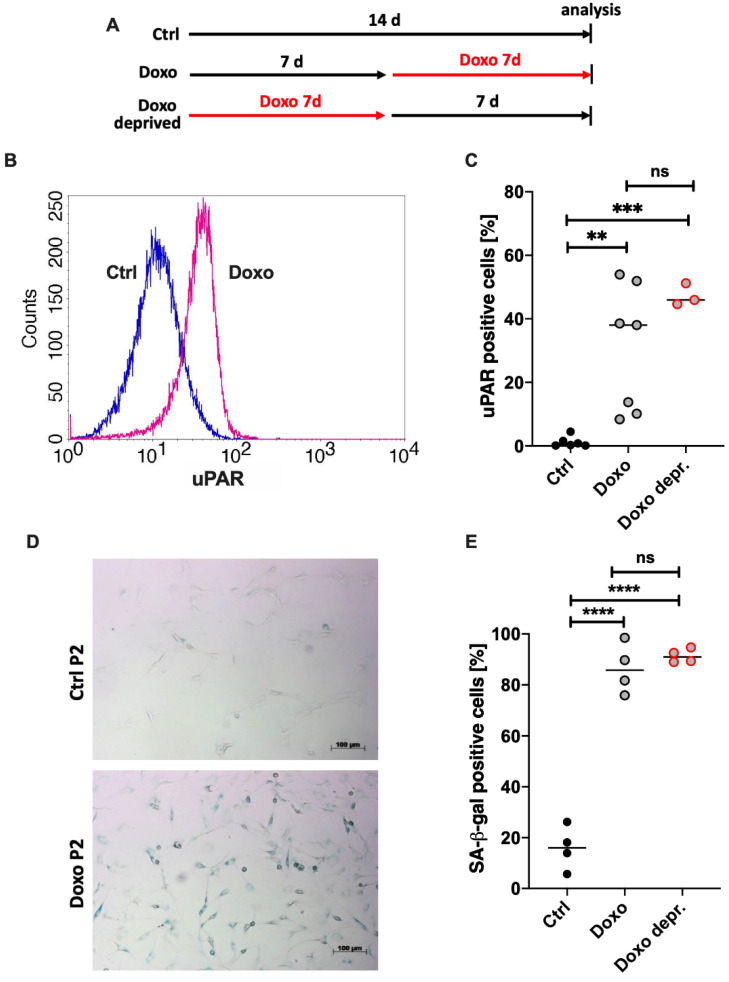
Impact of Doxo stimulation on uPAR expression and SA-β-gal activity in hAC. (**A**) Outline of the experimental setup. (**B**) Histogram of cytometric analysis of uPAR (CD87) on hACs unstimulated (blue) or stimulated with 0.1 µM Doxo for 7 d (pink). (**C**) Corresponding statistics of cytometric analysis of uPAR w/or w/o Doxo stimulation. (**D**) Exemplary phase contrast microscopy images of SA-β-gal staining in hACs (w/or w/o Doxo stimulation). (**E**) Corresponding quantification of SA-β-gal positive cells. Data are presented as scatter plot with median. Significant differences between groups are depicted as: **** *p* ≤ 0.0001; ***p ≤ 0.001; **p ≤ 0.01. Statistical analysis: (**C**,**E**) one-way ANOVA, Sidak’s multiple comparisons test. Ctrl = control (unstimulated cells), ns = not significant.

Cellular senescence is considered as an irreversible cell cycle arrest, indicating a certain stability of the respective phenotype. As in vitro studies of chondrosenescence might be affected by the presence of Doxo, maintenance of SIPS in hAC after the removal of Doxo was investigated in the following analyses using a deprivation regimen outlined in Figure 5A. 

uPAR (CD87) and SA-β-gal have been described as crucial markers in snCs including osteoarthritic hAC. Doxo stimulation resulted in a clear augmentation of uPAR-positivity in hAC compared to the unstimulated control (Figure 5B). The corresponding statistical analysis (Figure 5C) confirmed a significant increase of uPAR expression for the Doxo-stimulated cells (mean positivity: 30%; *p* = 0.0045) in comparison to the unstimulated control (mean positivity: 1%). Seven days after deprivation of Doxo, uPAR expression maintained upregulated. Moreover, Doxo stimulation resulted in enhanced levels of SA-β-gal activity as determined by means of a SA-β-gal staining, exemplarily shown in Figure 5D. Accordingly, the percentage of positive cells was significantly increased from 16% in unstimulated cells to 86% in Doxo-stimulated cells (*p* ≤ 0.0001; Figure 5E). In line with the findings of the uPAR expression, SA-β-gal activity was not decreased after deprivation of Doxo. 

**Figure 6:**
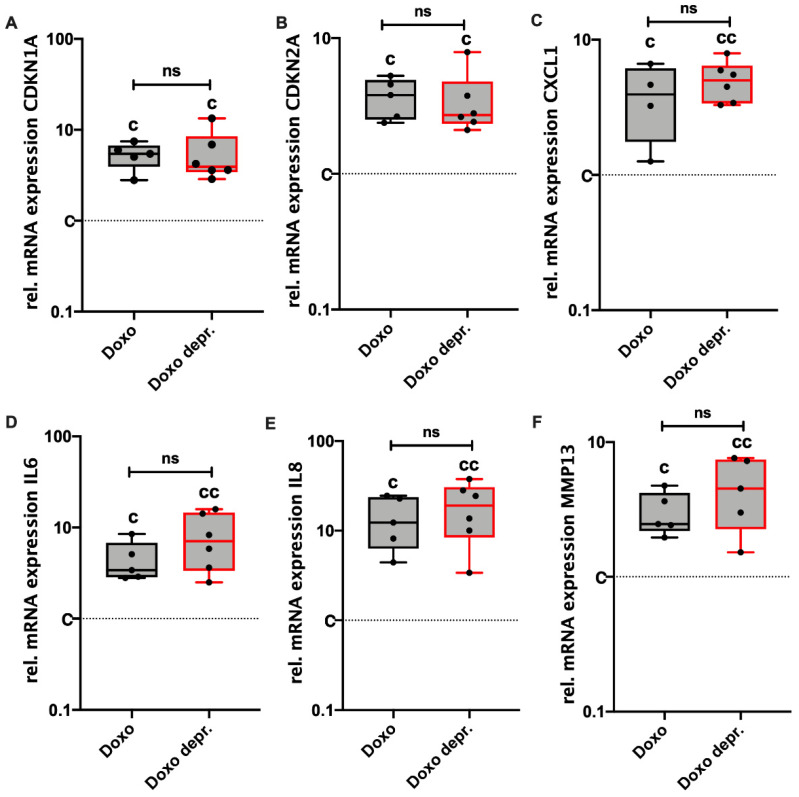
Continuing effect of Doxo stimulation on the gene expression of SA markers. Gene expression levels of (**A**) CDKN1A, (**B**) CDKN2A, (**C**) CXCL1, (**D**) IL6, (**E**) IL8, and (**F**) MMP13 were analyzed in hAC stimulated with 0.1 µM Doxo for 7 d. Doxo = analysis directly after 7 d of Doxo stimulation; Doxo depr. = analysis 7 d after deprivation of Doxo. Data are presented as box plots with median, whiskers min to max. Significant differences relative to unstimulated hAC are depicted as: cc = *p* ≤ 0.01; c = *p* ≤ 0.05. Statistical analysis: (**A**–**F**) one-way ANOVA, Sidak’s multiple comparisons test. Ctrl = control (unstimulated cells), rel. = relative, ns = not significant.

**Figure 7:**
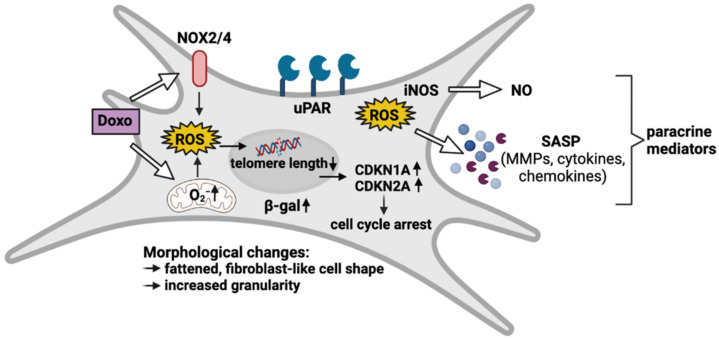
Overview of Doxo-mediated SIPS in hAC. Doxo treatment leads to oxidative stress by enhanced expression of ROS-generating NADPH oxidases (NOX2/4) and disturbance of mitochondrial function. Subsequent DNA damage results in upregulation of CDKN1A and CDKN2A, which initiate cell cycle arrest. ROS accumulation further triggers the expression of NO-generating iNOS and SASP factors. These paracrine mediators create a pro-inflammatory and catabolic microenvironment, not only fueling the progression of cartilage degeneration but also the spread of senescence. Moreover, Doxo-treated hAC express high levels of membrane-associated uPAR and intracellular β-gal, which are both well-described markers in chondrosenescence. Morphologically, senescent hAC are characterized by a flattened, fibroblast-like cell shape and an increase in granularity.

Maintenance of the senescent phenotype in hAC after deprivation of Doxo was further confirmed by means of gene expression analysis of different SASP markers. In fact, no clear alteration could be observed in the mRNA levels of CDKN1A, CDKN2A, CXCL1, IL-6, IL-8, and MMP-13, which were still significantly enhanced in the deprived approaches (Figure 6A–F). Overall, it was concluded that Doxo-induced SIPS remained stable for at least another 7 d after the deprivation of Doxo. An overview of Doxo-mediated effects on hAC is provided in Figure 7.

## 4. Discussion

As the most common degenerative joint disease, OA is of high medical relevance [26]. Although the incidence of OA is significantly increased in age, cartilage degeneration can also be provoked by traumatic joint injuries, resulting in posttraumatic OA. [3]. It is well known that aging and mechanical stress exhibit promoting effects on the imbalance between ROS production and the cellular anti-oxidant system in chondrocytes, resulting in chondrosenescence and progressive breakdown of cartilage matrix [13,27,28]. In fact, senescent chondrocytes have been found to be present at high numbers in human OA cartilage and were characterized by a dysfunctional behavior, comprising excessive production of ROS, proinflammatory cytokines, and chemokines. Overall, cellular senescence seems to play a pivotal role in the pathogenesis of both age- and trauma-related OA [3,23,28]. 

For the past three decades, Doxo, a natural anthracycline antibiotic, has been used as one of the most common chemotherapeutic drugs for the treatment of solid tumors, leukemias, and lymphomas [29]. However, it has been assumed that Doxo might be involved in the development of OA by inducing chondrocyte inflammation, cell cycle arrest, and/or apoptosis [25,30]. Doxo mediates apoptosis via alteration of DNA including mechanisms such as DNA intercalation, disruption of DNA repair by inhibition of type IIA topoisomerases, and ROS production [31]. Although it has been shown that Doxo treatment can induce DNA damage and apoptosis in rapidly dividing cells in vivo and in vitro, the cellular response to Doxo can differ within a population of cells. Accordingly, a recent study demonstrated that proliferative epithelial cells either underwent apoptosis or became senescent, as confirmed by the expression of the characteristic SA marker and an increase in cell size [32]. Molecular mechanisms, which are involved in Doxo-induced senescence comprise p53 upregulation, oxidative stress, and concurrent caspase inhibition [29]. Although Doxo-based in vitro models of cellular senescence have been established in various cell types [21,22,33], the specific properties of Doxo-mediated SIPS have not been precisely described so far, particularly with respect to hAC. Regarding the dose-dependent effects of Doxo to either induce apoptosis or senescence [33], we tested different concentrations of Doxo in hAC. Through this experiment, it could be demonstrated that hAC did not undergo apoptosis but became senescent at a dosage of 0.1 µM Doxo. Even though the cell count was significantly reduced, the apoptosis-associated caspase-3/7 activity remained unaffected. Subsequent cell cycle and gene expression analysis of Doxo-treated hAC confirmed that the reduction in cell count could be ascribed to CDKN1A (p21)- and CDKN2A (p16)-regulated cell cycle arrest. Mechanistically, we assumed that Doxo-mediated induction of cyclin-dependent kinase inhibitors might be caused by telomere shortening. In accordance with our findings, Li et al. described significant shortening of telomeres in normal human T lymphocytes and fibroblasts after Doxo stimulation, which was associated with lower telomerase activity, reduced expression of telomerase reverse transcriptase (hTERT), and telomere binding proteins as well as telomere dysfunction [34].

In the literature, several features of cellular senescence are well-described and almost ubiquitous in many cell types [35]. In line with these commonly defined hallmarks of cellular senescence, we observed an increase in CDKN1A and CDKN2A expression, accumulation of SA-β-gal positive cells, and alterations in cell morphology (Figure S3) in hAC after Doxo treatment. These SA markers have also been detected in cartilage as well as isolated hAC of OA patients, which exhibited a senescence-like phenotype indicated by a fibroblast-like or flattened morphology [3,34,35]. Furthermore, we noticed an increase in cell granularity, which, in fact, is considered as another criterion of cellular senescence [16]. It has been suggested that an increased size and number of lysosomes leads to a higher granularity in snCs, though the reason for this process has not been completely understood yet [36,37].

It is commonly assumed that snCs including chondrocytes and synoviocytes seem to fuel the development of classical OA hallmarks [8,38,39]. In line with this, we observed that Doxo treatment enhanced the secretion of several SASP markers including CXCL1, IL-8, and IL-6 in hAC. These pro-inflammatory SASP factors are associated with the pathophysiology of OA. CXCL1, for instance, seems to augment the expression of IL-6 in OA synovial fibroblasts, thus aggravating the inflammatory state in the joints [38,39]. Moreover, IL-8 and CXCL1 are considered as key mediators in phenotypical alteration of chondrocytes including hypotrophy and senescence [1,40], and have been found to be involved in the stabilization of SA growth arrest [8]. In addition, IL-6- and IL-8-mediated loss of the chondrogenic phenotype is associated with increased production of MMPs as well as ADAMTS4/5, which are also considered as SASP factors and drive enzymatic cartilage degradation [39,41]. These findings support the assumption that snCs promote the senescence of neighboring cartilage cells by release of SASP factors in a paracrine manner [8,42]. 

The expression of MMP-13 and ADAMTS-4/-5 is not only induced by cytokines but also by oxidative stress–mediators, which are both distinctive in chondrosenescence [3,17]. Diekman et al. described that increased levels of p16 correlated with the expression of SASP factors such as MMP-1, MMP-13, and IGFBP3 in hAC. However, neither in vitro silencing nor somatic inactivation of p16 in vivo could prevent senescence and subsequent expression of SASP factors in chondrocytes and thus OA progression. Therefore, it was concluded that the secretion of SASP factors rather than p16 activity promotes chondrocyte senescence in OA [43].

Overall, it has been suggested that matrix degradation products may amplify the synovial inflammation, which in turn promotes the production of MMPs. Consequently, matrix degeneration stimulates synovial inflammation and vice versa, creating a vicious cycle [44,45]. 

Transmembraneous uPAR promotes the formation of active plasmin and thus represents an important player in the MMP activation cascade [46]. Accordingly, uPAR expression has been associated with cartilage degradation and was found to be enhanced on synovial cells, in synovial fluid, and also in the plasma of OA and RA patients [47]. Previous studies have reported that uPAR expression on chondrocytes is upregulated in response to cytokines and mechanical stress [46,48]. Overall, uPAR seems to be increased in snCs and chondrocytes from OA patients, and has thus been considered as a potential SA marker [46,47,48] Our results demonstrate that the basal expression of uPAR on hAC isolated from macroscopically intact OA tissue is very low but significantly upregulated during Doxo-mediated SIPS. This finding implies that senescent chondrocytes not only secrete excessive amounts of MMPs, but might also promote the activation of these catabolic enzymes.

According to the literature, oxidative stress results from excessive ROS generation and has not only been shown to play a pivotal role in the pathology of OA, but has also been considered as one of the major drivers of cellular senescence, and in particular, SIPS [25,49,50,51]. Kang et al. previously demonstrated the induction of SIPS by ROS after administration of Doxo in primary murine and human articular chondrocytes [25].

To further investigate the mechanisms of Doxo-mediated oxidative stress and mitochondrial dysfunction in hAC, we evaluated the expression of different NADPH oxidases and enzymes of the cellular antioxidant system as well as ROS generation. Along with the mitochondria, NOXs are the main sources of ROS within cells [52]. The significantly enhanced expression of NOX2 and NOX4 indicated that ROS production might be increased in Doxo-treated hAC, while CAT, SOD1, SOD2, and SIRT1, as part of the cellular antioxidant defense system, showed no alteration in RNA expression. However, exemplary protein analysis indicated that SIRT1 and SOD2 expression might be increased in response to Doxo-mediated oxidative stress to a certain extent. Previously, Doerr et al. reported that intramuscular injection of Doxo reduced the mRNA levels of SOD2 and glutathione peroxidase 1 (GPX1), while that of SOD1, CAT, and SIRT1 was not affected in soleus muscle cells. Despite the decline in some enzymes on gene expression level, none of the tested enzymes was suppressed on the protein level, as determined by western blot analysis [53].

In line with the gene expression analysis, DCFDA and MitoSOX assays confirmed a significant accumulation of intracellular and mitochondrial ROS, respectively, in Doxo-treated hAC. In fact, Doxo is known to induce mitochondrial dysfunction by binding cardiolipin in the inner mitochondrial membrane, thus disturbing the electron transport chain [54]. Previous studies have reported that ROS produced by NOX2 and NOX4 as well as mitochondria can activate signaling cascades that induce senescence in OA chondrocytes [55,56,57]. Taken together, our results imply that Doxo-mediated SIPS might be triggered by enhanced production of ROS, while the cellular antioxidant system does not seem to be impaired. Since OA-associated chondrosenescence has also been ascribed to mitochondrial dysfunction and oxidative stress, Doxo-mediated SIPS might represent a more appropriate approach to study senescence in hAC than, for instance, irradiation-based models.

Furthermore, the present study confirmed that Doxo not only provokes intracellular ROS accumulation but also gene expression of iNOs and subsequent NO release by hAC. Under physiological conditions, NO has been identified as an important second messenger involved in the regulation of cartilage degradation [58]. Moreover, NO has also been demonstrated to induce apoptosis in OA synoviocytes and chondrocytes by activating the expression of pro-apoptotic caspase-3 and caspase-9 [59]. In the context of cellular senescence, NO is thought to act as a modulator of various SASP pathways and as an inducer of SIPS due to direct or indirect DNA damage, mediated by its highly reactive derivatives such as peroxynitrites [60,61]. 

In the literature, senescence has been described as an irreversible cell cycle arrest [62]. To confirm irreversibility of the senescent state, we assessed the expression of SA markers in hAC after the deprivation of Doxo. Seven days after the removal of Doxo, uPAR expression and SA-β-gal activity as well as the gene expression of SASP-associated markers, maintained significantly upregulated in the deprived group. Moreover, morphological alteration was even more pronounced after removal of Doxo (Figure S3), which was also observed for the before mentioned markers by trend. In fact, results from previous studies using HeLa cells imply a time delay effect in Doxo-mediated senescence, as demonstrated by increased SA marker expression six days after removal of the chemotherapeutic drug [32]. 

Taken together, our findings confirmed that the senescent phenotype in Doxo-treated hAC persisted for at least seven days under drug-free conditions, and might even be enhanced by this procedure. This is an important aspect to study in the characteristics of snCs and the underlying mechanisms in vitro, particularly, in terms of studies addressing the paracrine effects on non-senescent cells, in which residues of Doxo within the conditioned medium would influence the outcome.

## 5. Conclusions

Together, our findings affirm that Doxo treatment causes a SIPS-like phenotype in hAC, corresponding to previous studies in other cell lines. Senescence was demonstrated by characteristic hallmarks such as SASP marker expression and cell cycle arrest (Figure 7). Furthermore, the deprivation of Doxo confirmed the persistence of SA characteristics in hAC, which allows for subsequent analysis without the addition of the drug. Overall, this study proves the suitability of Doxo to induce and evaluate SIPS in hAC in vitro. 

## Data Availability

Data are contained within the article.

## Supplementary Materials

The following are available online at https://www.mdpi.com/article/10.3390/cells11071106/s1. Figure S1: Gating strategy of the flow cytometric analysis. Figure S2: Quantitative evaluation of exemplary western blot analysis and corresponding gels. Figure S3: Morphological alteration of isolated hAC affected by osteoarthritis and after Doxo stimulation, respectively.
