# Supplementary material for: In Vitro Characterization of Doxorubicin-Mediated Stress-Induced Premature Senescence in Human Chondrocytes
Source: Cells. 2022 Mar 25;11(7):1106. doi: 10.3390/cells11071106 (PMC8998002; doi:10.3390/cells11071106)

Figure S2: Quantitative evaluation of exemplary western blot analysis and corresponding gels. SIRT1 (A, B) and SOD2 (C,D) of hAC 7d after 0.1 µM Doxo stimulation. Ctrl= control (unstimulated cells), M= marker/ protein standard.

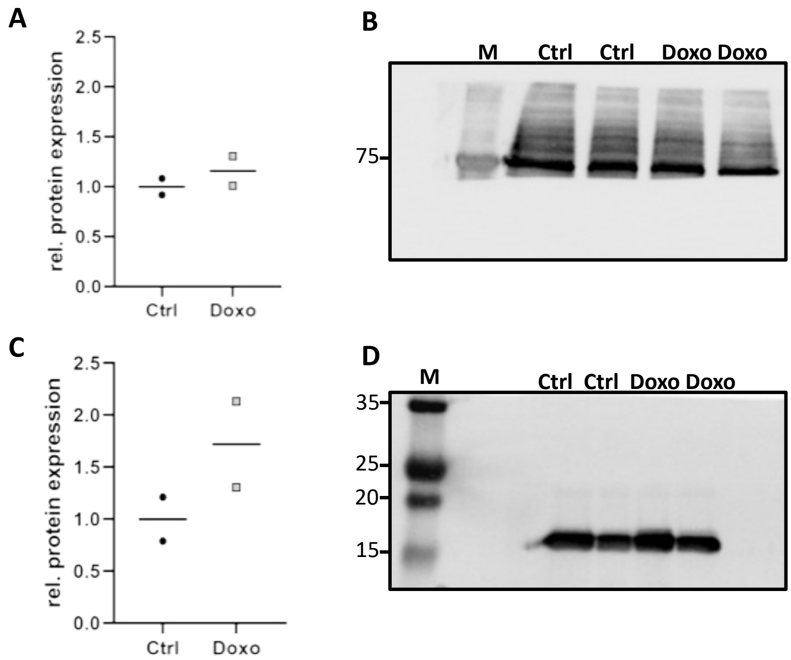

Supplement: Supplementary file 1 [file cells-11-01106-s001.zip › Figure S2_Doxo.pdf]
