# Supplementary material for: In Vitro Characterization of Doxorubicin-Mediated Stress-Induced Premature Senescence in Human Chondrocytes
Source: Cells. 2022 Mar 25;11(7):1106. doi: 10.3390/cells11071106 (PMC8998002; doi:10.3390/cells11071106)

Figure S3: Morphological alteration of isolated hAC affected by osteoarthritis and after Doxo stimulation, respectively.

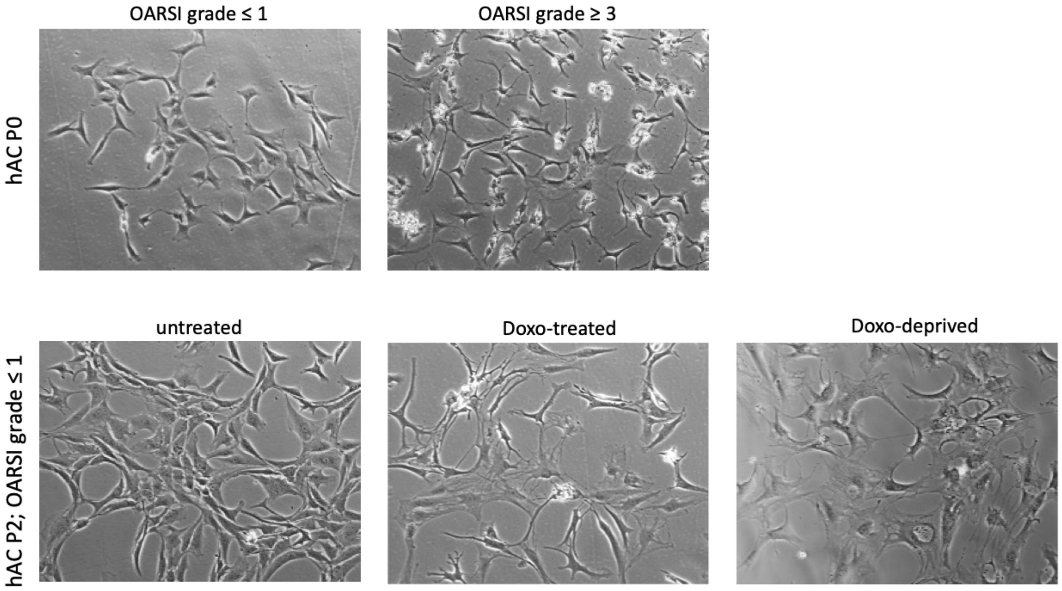

Supplement: Supplementary file 1 [file cells-11-01106-s001.zip › Figure S3_Doxo.pdf]
